# Decoupling Backward Scattering Control and Forward Beamformed Communication in a Shared‐Aperture Retro‐Reflective/Transmissive Metasurface

**DOI:** 10.1002/nap2.70226

**Published:** 2026-07-28

**Authors:** Shulei Zhang, Ruichao Zhu, Yuxiang Jia, Fan Wu, Xinqi Cai, Zhihui Zhang, Dan Liu, Yuxiao Guo, Jiafu Wang

**Affiliations:** ^1^ Shaanxi Key Laboratory of Artificially‐Structured Functional Materials and Devices Air Force Engineering University Xi'an Shaanxi China; ^2^ Key Laboratory for Soft Chemistry and Functional Materials of Ministry of Education, School of Chemistry and Chemical Engineering Nanjing University of Science and Technology Nanjing Jiangsu China

**Keywords:** beamforming, metasurfaces, retroreflection, scattering enhancement, transmission

## Abstract

Integrating electromagnetic scattering engineering and high‐quality wireless links on the same platform is fundamentally challenged by the coupling between reflection and transmission channels in shared‐aperture structures. Here we report a programmable retro‐reflective and transmissive metasurface (RRTM) that decouples backward scattering enhancement from forward beamformed communication within a single co‐aperture. A four‐state meta‐atom using two embedded positive–intrinsic–negative (PIN) diodes provides 1‐bit phase control in both reflection and transmission channels, enabling unified digital coding for dual functionalities. A 16 × 16 prototype is driven by a field‐programmable gate array (FPGA) and cascaded shift registers for rapid reconfiguration among transmissive beam steering, retro‐reflective scattering enhancement and hybrid operation. Experiments at 8 GHz validate wide‐angle transmit beam steering up to ± 60° and angular‐adaptive retroreflection up to 50° incidence. A video transmission link with a well‐clustered 16‐ary quadrature amplitude modulation constellation further confirms the quality of beamformed communication. Finally, a checkerboard co‐aperture coding strategy enables simultaneous retroreflection and transmit beam scanning, preserving narrow beams and low sidelobes in concurrent operation. The proposed architecture provides a practical route toward integrated platforms that require coordinated scattering control and directional wireless links.

## Graphical abstract

Conceptual illustration of the retro‐reflective and transmissive metasurface (RRTM). The blue radar establishes communication with the UAV via the transmissive mode, whereas the purple surveillance radar acquires the status of friendly units through the retro‐reflective mode and the two modes operate simultaneously within the same decoupled aperture.

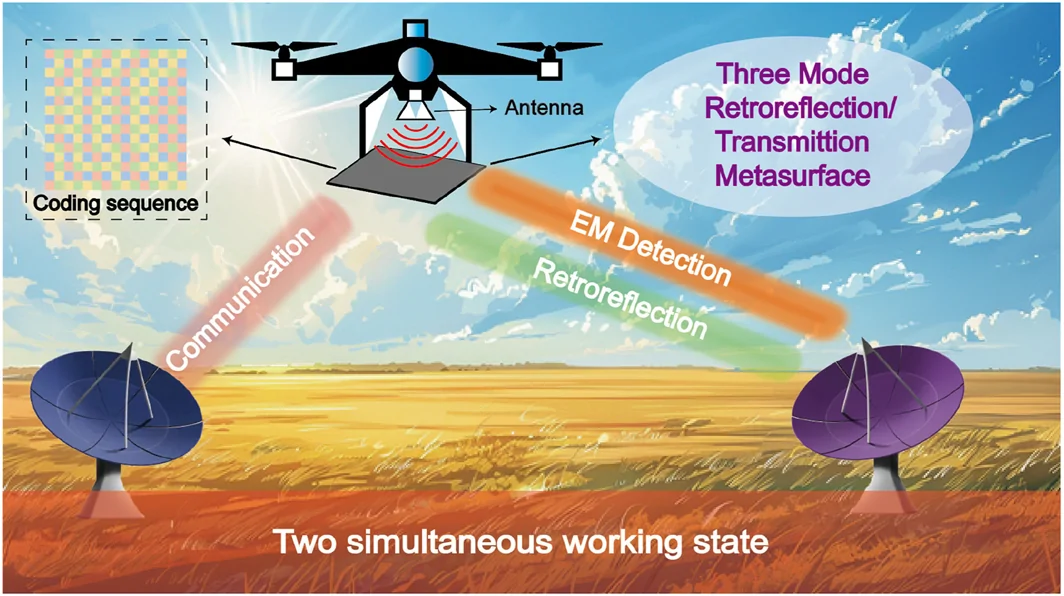

## 1 Introduction

Emerging airborne and mobile platforms increasingly demand two seemingly conflicting electromagnetic capabilities: (i) engineered backward scattering responses for sensing, tracking, signature management or retroreflective backscatter enhancement, and (ii) high‐quality forward wireless links for reliable connectivity and information delivery [1, 2]. Achieving both capabilities within the same size, weight, power and aperture budget is challenging, because scattering engineering and beamformed communication typically compete for the same physical aperture and controllable degrees of freedom. Traditional implementations rely on spatially separated apertures—dedicated phased arrays [3, 4] for beamforming and passive corner reflectors or Van Atta arrays for retroreflection [5, 6, 7, 8]—incurring severe penalties in payload weight, aerodynamic drag and electromagnetic interference. A shared‐aperture solution that can dynamically switch between these scattering regimes while maintaining transparency for independent transmission functions is therefore highly desirable, yet fundamentally challenged by conflicting electromagnetic boundary conditions [9, 10].

Metasurfaces provide a compact route to manipulation of amplitude/phase/polarisation [11, 12, 13, 14, 15], offering an attractive candidate for shared‐aperture scattering‐and‐link integration. Programmable metasurfaces have enabled reconfigurable wavefront control, yet existing approaches often emphasise only one side of this requirement [16, 17, 18]. Reflection‐type programmable metasurfaces and reconfigurable intelligent surface (RIS) architectures are effective for controlling backward scattering but do not inherently provide a forward beamformed link without additional radiating hardware [19, 20, 21, 22]. Conversely, transmissive or radiating aperture designs can support directional links but typically leave backward scattering behaviour uncontrolled [23, 24, 25]. Transmission–reflection integrated (TRI) and Janus‐type metasurfaces provide a promising route toward shared‐aperture full‐space control [26, 27].

The authors in reference [28] proposed and demonstrated a reconfigurable bi‐directional spatially‐fed array that ingeniously integrates the functionalities of reconfigurable transmitarray (TA), reflectarray (RA), and transmit‐reflectarray (TRA). By integrating PIN diodes in the intermediate layer, it can dynamically switch between acting as a polarisation conversion layer or a polarisation‐preserving layer. Combined with a crossed grating as a polarisation‐selective layer, it achieves 1‐bit dynamic phase control over reflected and transmitted waves. Through meticulously designed antenna transceiver architectures along electromagnetic wave propagation paths—incorporating receiving layers, vias, ground isolation layers and radiating layers—the state control of PIN diodes enables precise phase modulation for both transmitted and reflected waves [26, 29, 30, 31]. This integrated design holds significant application potential in indoor communications. However, within the broader scope of integrated air‐to‐ground (ATG) communication and sensing, stealth and enhanced backscattering are equally crucial considerations. In particular, concurrent operation is frequently realised by contiguous aperture partition, which reduces effective aperture for each task and degrades beam quality of both functions.

In this work, we target a practical and verifiable objective: decoupled coexistence of backward scattering engineering and forward beamformed communication in a single shared‐aperture metasurface. Rather than claiming zero‐loss simultaneity, we explicitly recognise that concurrent multi‐functionality introduces a quantifiable trade‐off, and we aim to provide an architecture that supports single‐channel optimal operation when needed, and concurrent operation without relying on contiguous aperture shrinkage.

To this end, we develop a programmable retro‐reflective/transmissive metasurface based on a four‐state PIN‐loaded meta‐atom. States ‘00/11' establish a π‐phase pair in reflection (enabling retroreflection/backscatter enhancement), whereas states ‘01/10’ create a π‐phase pair in transmission (enabling beam steering). A 16 × 16 prototype driven by an FPGA and cascaded shift registers supports rapid switching among transmissive beam steering, angular‐adaptive retroreflection/backscatter enhancement and concurrent operation. Experiments around 8 GHz demonstrate transmissive beam steering over a wide angular range (up to ± 60°) and backward retroreflection/backscatter enhancement under oblique incidence (up to 50°). A beamformed wireless link is further verified by a well‐clustered 16‐QAM constellation, confirming preserved modulation quality under programmable beam steering. For concurrent functionality, we introduce an interleaved (checkerboard) co‐aperture coding strategy—an interleaved spatial‐division multiplexing (SDM) approach—that enables simultaneous transmission and retroreflection with acceptable loss, with an explicitly quantified performance trade‐off relative to single‐channel optimal cases.

## 2 Results and Discussion

Figure 1 presents a schematic diagram of the proposed simultaneous retro‐reflective and transmissive metasurface, integrated onto a UAV and configured with a defined coding sequence. The metasurface operates in two distinct modes: reflection and transmission. Within each mode, two states with a 180° phase difference are available, serving as phase shifters for beam steering. A horn antenna, located above the metasurface, functions as its feed, facilitating directional communication in the transmissive mode. In the reflective mode, the metasurface can either diminish radar cross section (RCS) through randomised coding or augment backscattering via a phase gradient. The depicted functionality in the figure is retroreflection. When all four coding states coexist, the metasurface can concurrently engage in communication with blue authorised receivers in the transmissive mode, and simultaneously achieve surveillance by authorised receivers or provide jamming and camouflage against external surveillance radar through the retro‐reflective mode. To enable these functions through a unit that simultaneously manipulates reflected and transmitted waves, a metasurface designed to operate on the same polarization is necessary. Consequently, a transceiver antenna structure is adopted, incorporating embedded PIN diodes for dynamic control of transmission or reflection and the implementation of phase shifting.

**FIGURE 1:**
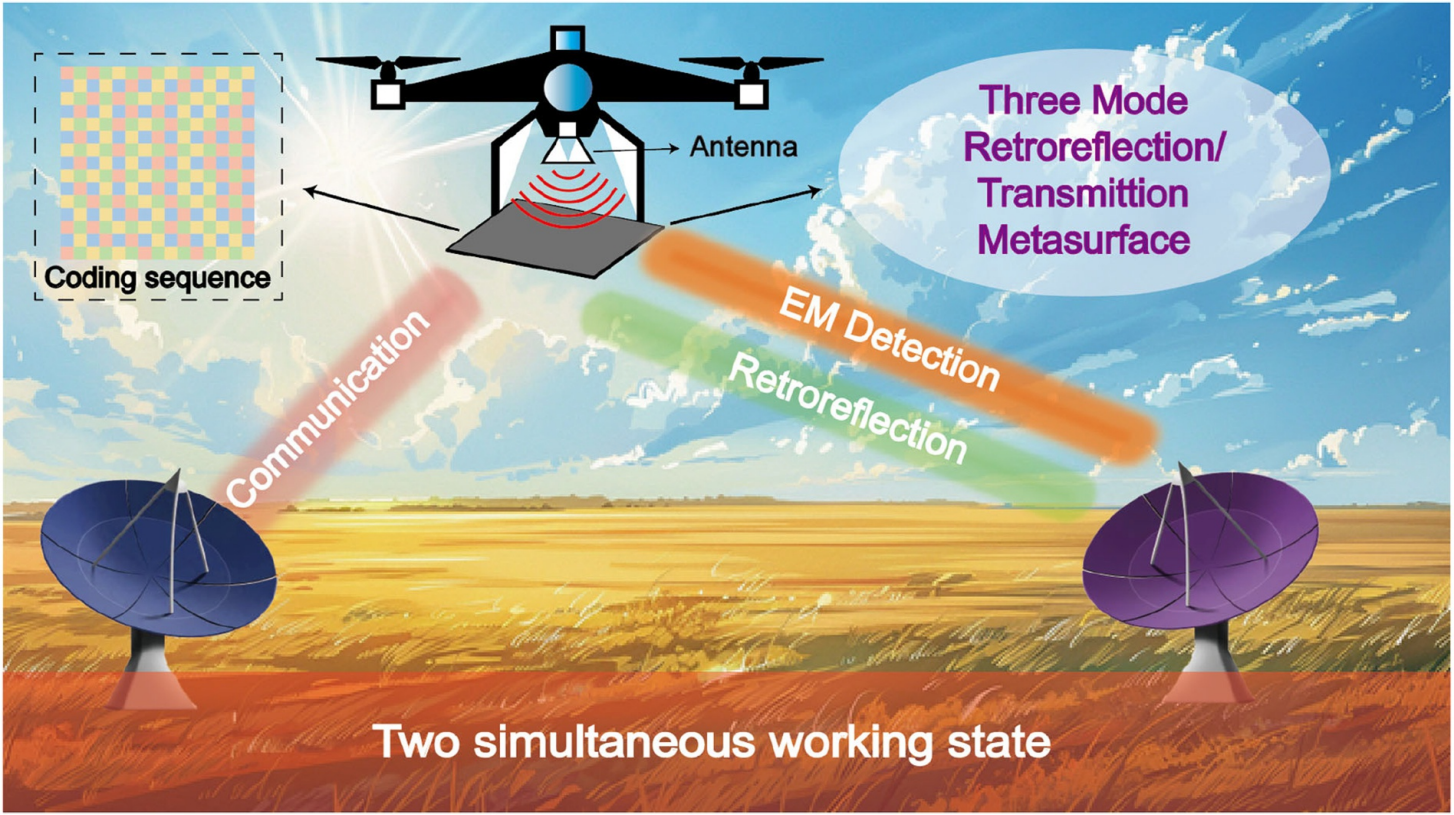
Conceptual illustration of the retro‐reflective and transmissive metasurface (RRTM). The blue radar establishes communication with the UAV via the transmissive mode, whereas the purple surveillance radar acquires the status of authorised receivers through the retro‐reflective mode and the two modes operate simultaneously.

### 2.1 Meta‐Atom Design

The metasurface element with integrated transmission–reflection functionalities supports full‐space operation. As illustrated in Figure 2a, the 3D‐rendered schematic demonstrates the partitioned architecture. The four‐layer metallic configuration and corresponding dimensional parameters are respectively presented in Figure 2b–e, featuring a compact and fabrication‐friendly structure.

**FIGURE 2:**
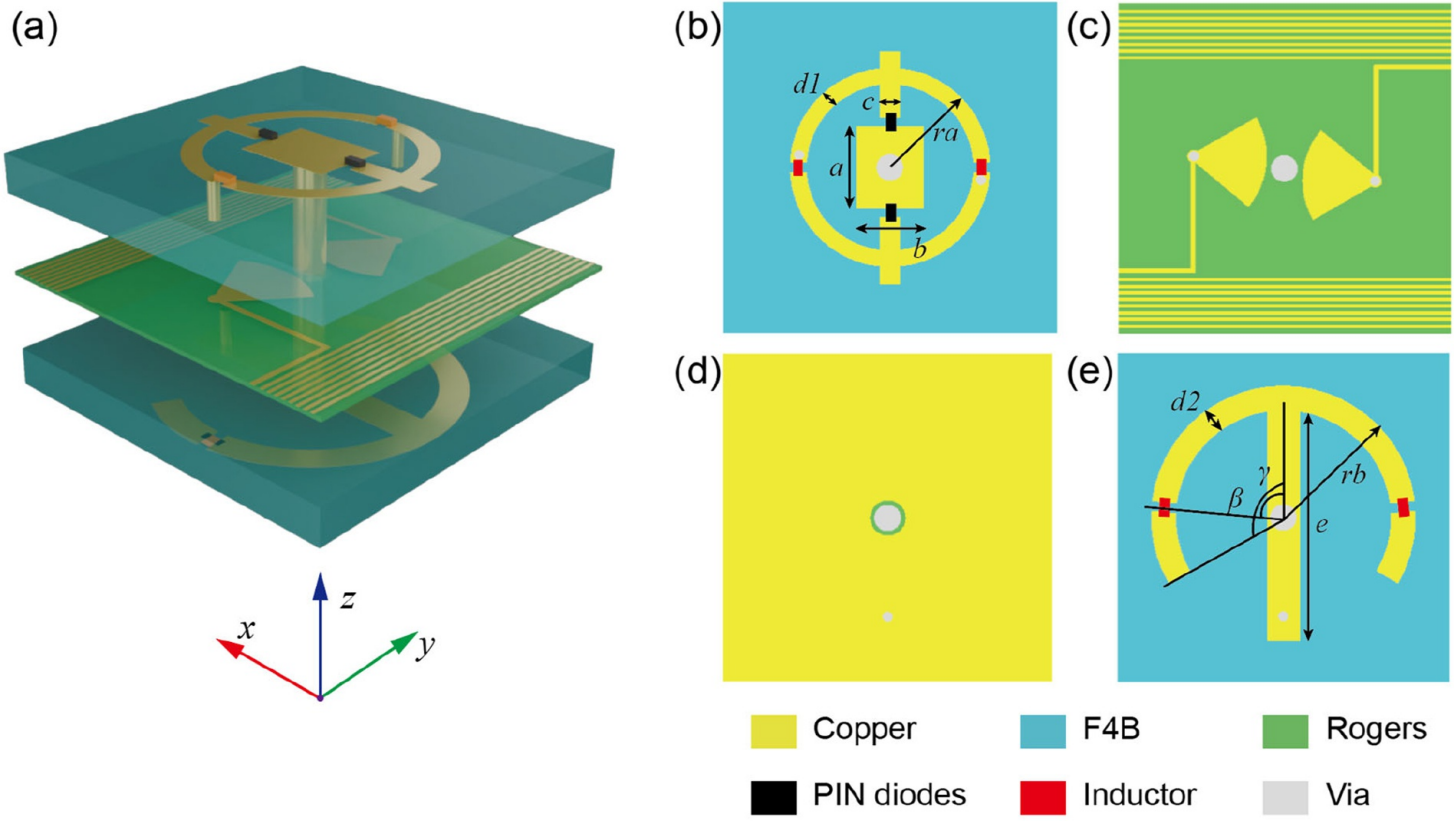
(a) Perspective 3D‐rendered view of the unit cell. (b) Top layer. (c) Bias layer. (d) Ground layer. (e) Bottom layer.

The top layer comprises a central patch with length *a* and width *b*, flanked by two arc structures at its ends. Two SMP1320‐079 PIN diodes are surface‐mounted at both ends of the patch with cathodes oriented toward the central metallic region. Blind vias extending from the arc terminals to the second metallic layer enable Vcc biasing voltage. Consequently, inductors are strategically placed in the red‐highlighted regions within the unit cell to isolate the DC bias from the RF signals.

The second layer houses the feeding network, whose influence on metasurface performance proves negligible due to electromagnetic shielding from both the third‐layer ground plane and fan‐shaped isolation segments. A through‐hole with a diameter of 1.6 mm interconnects the first and fourth layers, establishing a radio frequency signal transmission channel to the radiation port. The bottom arc structure (outer radius *rb*) incorporates inductors for resonant enhancement, effectively amplifying both transmission and reflection magnitudes. Detailed dimensional parameters are: *a* = 5 mm, *b* = 4 mm, *c* = 1.2 mm, *ra* = 6 mm, *e* = 14 mm, *rb* = 8 mm, *d*2 = 1.5 mm, *d*1 = 1 mm, *γ* = 85° and *β* = 120°. The diode switching equivalents exhibit RLC parameters of 0.5 Ω, 0.7 nH and 0.24 pF.

Under *z*‐axis wave incidence, the element demonstrates dual operational modes. Phase difference approximately 180° between modes is achieved through binary diode switching, as quantified in Table 1. The OFF state of a diode is encoded as ‘0’, and the ON state is encoded as ‘1’. The switching states of the two diodes are represented by two‐digit binary codes: ‘00’, ‘11’, ‘01’ and ‘10’. Reflection mode occurs at codes ‘00’ and ‘11’, exhibiting 180° phase difference near 7.7 GHz. Surface current analysis in Figure 3 reveals that cooperative resonance between top and bottom layers establishes this reflective configuration. Symmetric current distribution across the bottom layer suppresses radiation leakage, whereas path‐length variations between ‘00’/‘11’ states induce the observed phase difference of near 180°.

**TABLE 1:** PIN diode states and corresponding operation modes.

Encoding	PIN states of P1 and P2		Mode state	Phase
00	OFF	OFF	Reflection	Rf1
11	ON	ON	Reflection	Rf1 + π
01	OFF	ON	Transmission	Tf1
10	ON	OFF	Transmission	Tf1 + π

**FIGURE 3:**
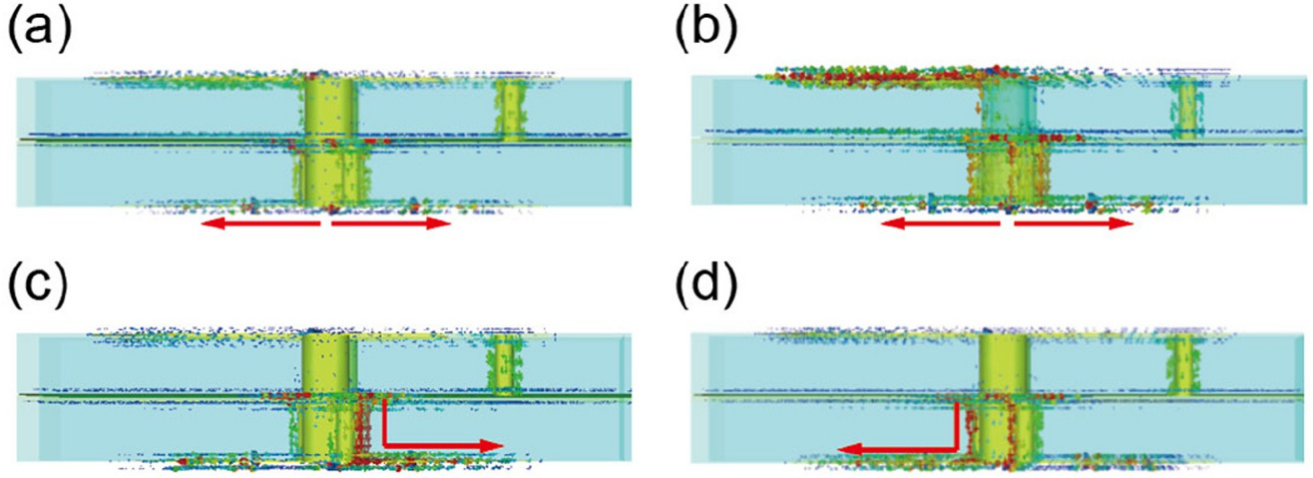
Surface current distribution at 7.7 GHz for reflection states ‘00’ (a) and ‘11’ (b). Surface current distribution at 7.7 GHz for transmission states ‘01’ (c) and ‘10’ (d).

Conversely, transmission mode activates under ‘01’/‘10’ coding where asymmetric diode states (one OFF, another ON) enforce unidirectional current propagation. This asymmetric current distribution forms a radiation‐enabling structure, facilitating RF signal reception through the feeding layer, via transmission and final radiation from the output layer. Maxwell equation analysis confirms that opposing surface current directions between ‘01’ and ‘10’ states generate complementary 180° phase relationships [32].

The theoretical framework of the dual‐mode metasurface is validated through full‐wave simulations in CST Microwave Studio. Periodic boundary conditions are imposed on lateral surfaces with open space along ± *z* directions, emulating infinitely periodic structures under plane wave illumination.

Figure 4a demonstrates the transmission characteristics under ‘01’/‘10’ coding states, exhibiting transmission magnitudes exceeding −3 dB within 7.2–8.2 GHz while maintaining reflection magnitudes below −10 dB. This confirms predominant energy transmission efficiency. The phase response in Figure 4b reveals a stable 180° phase difference induced by counter‐directed surface currents, enabling effective 1‐bit phase quantisation for beam manipulation.

**FIGURE 4:**
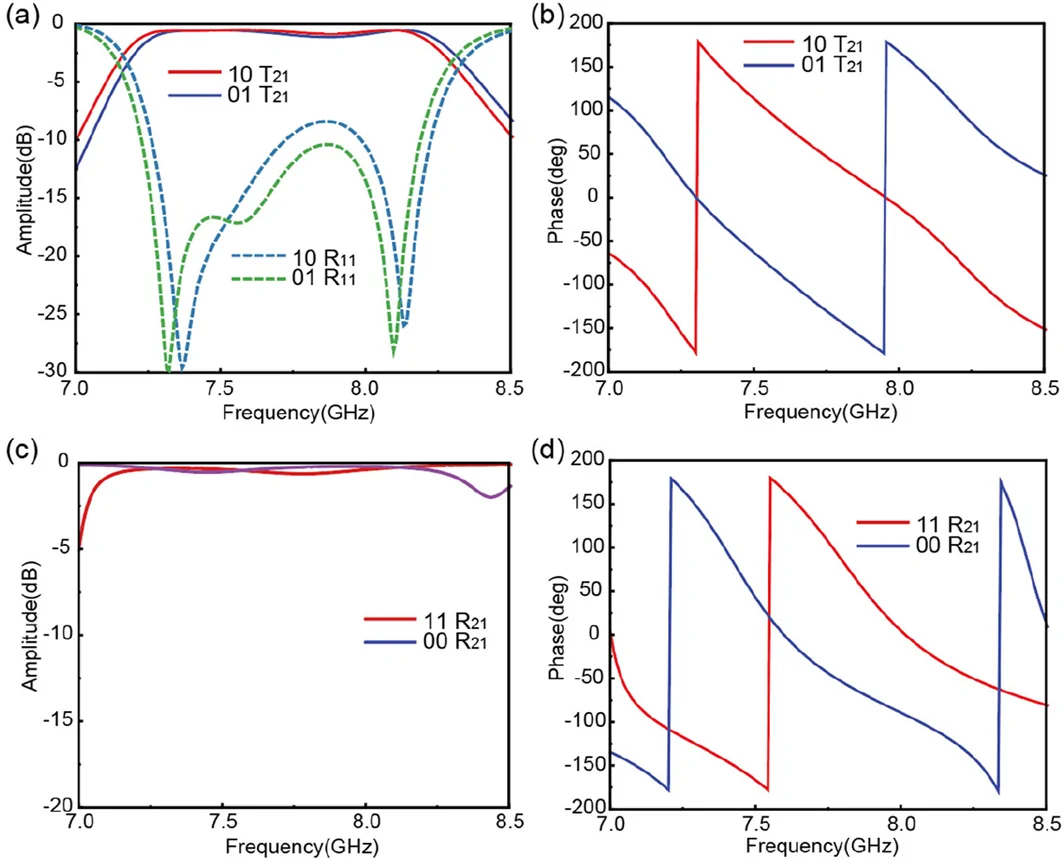
Transmission and reflection amplitude (a) and phase relationship of transmission (b) for states ‘01’ and ‘10’ in transmission mode. Amplitude (c) and phase (d) of states ‘00’ and ‘11’ in reflection mode.

Figure 4c,d present reflection‐mode performance for ‘00’/‘11’ states, showing reflection magnitudes within −3 dB across the 7.2–8.2 GHz bandwidth. The phase difference between ‘00’ and ‘11’ states remains within 180° ± 30° consistency over 7.4–7.7 GHz, attributable to resonance‐dependent current path variations. Notably, this configuration achieves simultaneous 1‐bit phase control in both reflection/transmission modes using only two diodes, significantly reducing power consumption and feed network complexity for scalable array implementations.

Besides, the co‐polarised reflection amplitudes are less than −9 dB, whereas other components are less than −15 dB, also demonstrating good isolation. Therefore, at the unit cell level, the isolation between the two functionalities can be considered good, providing the fundamental conditions for decoupling.

### 2.2 Transmission Mode Operation

The metasurface serves as an excellent and cost‐effective full‐duplex device. In its transmissive mode, it can be utilised for directional communication by modulating the radiation pattern of an electromagnetic wave emitted from a horn antenna. Alternatively, it can function as a receiving device, enabling directional reception of electromagnetic waves from various directions for information collection. A simulation of a 16 × 16 unit cell metasurface array was conducted. As shown in Figure 5a, a horn antenna was positioned 350 mm directly above the metasurface, employing a direct feed to illuminate the metasurface with a spherical wave. The horn aperture dimensions are 72 mm × 58 mm. The gain of the horn antenna is approximately 13.2 dB at 8 GHz.

**FIGURE 5:**
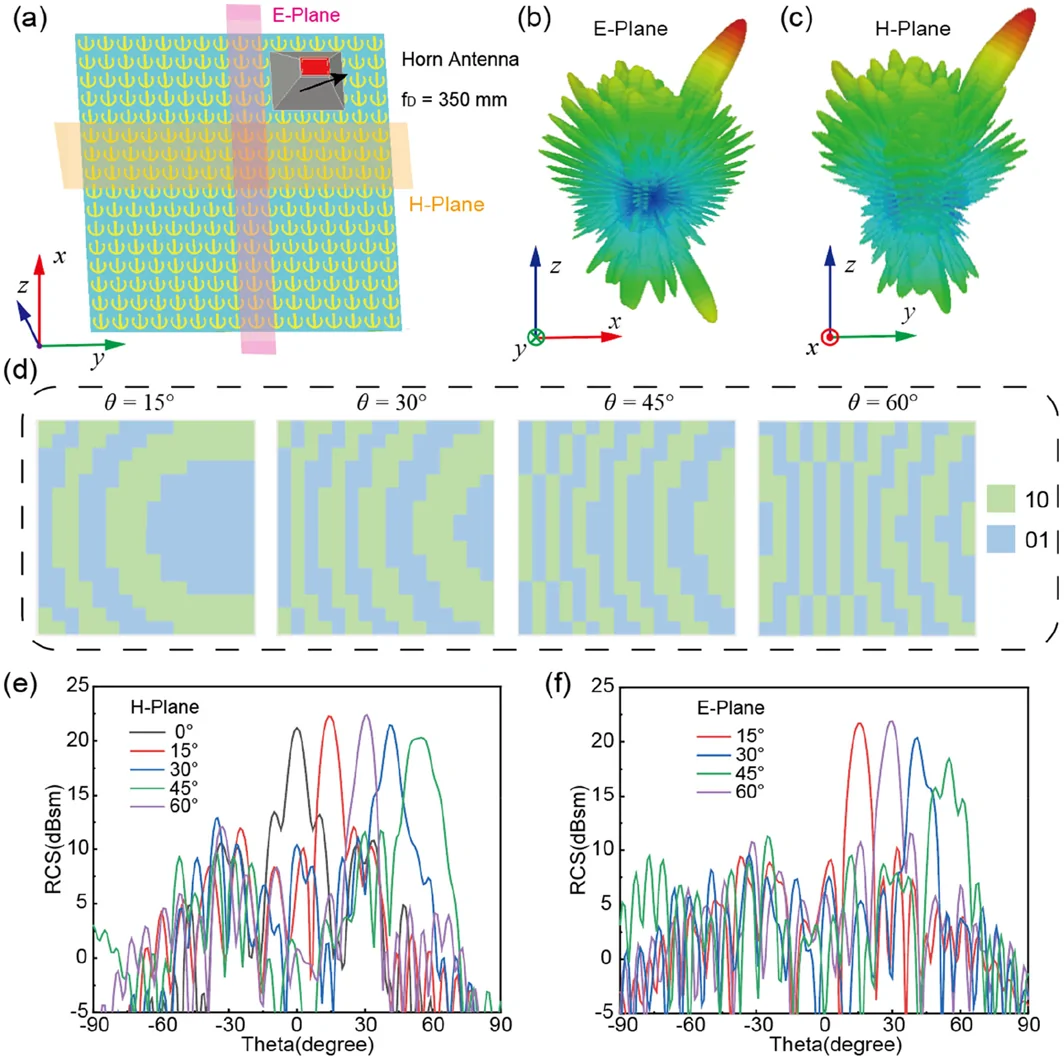
(a) Schematic diagram of the transmitarray and feed source positions during simulation. 3D radiation patterns when simulating at 30° in the E‐plane (b) and H‐plane (c). (d) Phase distribution images for different beam scanning angles (15°, 30°, 45°, and 60°) calculated using the reflection array theory. (e) Radiation pattern in the H‐plane at 8 GHz. (f) Radiation pattern in the E‐plane at 8 GHz.

For ease of subsequent testing, the CST simulation employed ‘open add space’ boundary conditions in all directions. Angle scans for both the E‐plane and H‐plane were simulated. A time‐domain solver was utilised, and a far‐field monitor was added to acquire far‐field gain radiation patterns. The phase distribution of the metasurface was calculated using the theory of reflectarray antennas [33, 34].

The ideal phase distribution $\varphi_{mn}$ for the element located in the *m*th row and *n*th column is as follows:

(1) $$\varphi_{mn}=k_{0}\left(\left|{\hat{r}}_{mn}-{\hat{r}}_{f}\right|-{\hat{r}}_{mn}\cdot \hat{u}\right)+\varphi_{0},$$

where ${\hat{r}}_{mn}$ is the element position vector, $k_{0}$ is the wave vector of the free space, ${\hat{r}}_{f}$ denotes the feed position vector, $\hat{u}$ indicates the unit vector of the desired reflected beam direction, $\varphi_{0}$ represents a constant reference phase.

Then the continuous ideal phase distribution should be quantised as two states using the following criterion:

(2) $$\varphi_{mn}=\left\{\begin{array}{l} 0{}^{\circ},0{}^{\circ}<\varphi_{mn}<180{}^{\circ}\\ 180{}^{\circ},\text{otherwise} \end{array}\right..$$

The aforementioned formulae enable the calculation of target phases for specific scanning angles at a given frequency, corresponding to the distribution matrices for ‘01’ and ‘10’ encodings. It is noteworthy that for continuous‐phase arrays, variations in reference phase only result in a global phase shift, thus not affecting the final simulation outcomes. However, in the context of 1‐bit quantisation, different reference phases lead to distinct 1‐bit phase distributions, consequently influencing the results. In this study, simulations were performed for a 30° scanning angle, and the impact of reference phase variations was found to be minimal. Therefore, for computational simplicity, the reference phase was set to 0 for all other scanning angles, without further optimisation.

Figure 5b,c present the 3D radiation patterns of the transmitted wave at a 30° scanning angle, as obtained from simulations. Figure 5d displays the encoding matrices calculated using the aforementioned formula for beam deflection angles θ of 15°, 30°, 45° and 60°. The two colours in the matrix represent the encodings for the ‘01’ and ‘10’ transmission modes. This encoding scheme is applied to both the E‐plane and H‐plane, with the only difference being a 90° rotation of the encoding matrix for the E‐plane. Figure 5e shows the simulation results for the H‐plane, demonstrating a gain exceeding 22 dB within 0°–45°. For a beam scanning angle of 60° in the H‐plane, the simulations predicted a 2 dB scan loss and less than 2° pointing error. Figure 5f, illustrating the E‐plane simulation results, maintains good performance less than 30°. However, performance deteriorates at larger angles, particularly at 45° and especially 60°. This degradation may be attributed to the insufficient angular stability of the E‐plane. More specifically, the observed degradation at large scan angles arises from the combined effects of: (i) the angular sensitivity of the meta‐atom itself under oblique incidence, (ii) spherical‐wave illumination and amplitude taper introduced by the nearby horn feed, (iii) 1‐bit phase quantisation error and (iv) finite‐aperture truncation and associated mutual‐coupling/edge effects. Nevertheless, overall, considering that the horn antenna and reference phase were not optimised, the metasurface is capable of achieving the scanning effects depicted in the figures with acceptable gain.

The metasurface sample, fabricated via printed circuit board (PCB) technology, consists of a 16 × 16 array of unit cells with a total area of 360 mm × 320 mm. The top (a) and bottom (b) of the fabricated sample are illustrated in Figure 6. There are two PIN diodes on each meta‐atom so in order to control the states of 512 PIN diodes, the bias layer incorporates 512 feed lines. Every 32 feed lines are soldered to a 32‐pin connector, which is then interfaced via flexible printed circuit (FPC) cables to the control board. The control board comprises 64 cascaded shift registers, enabling serial input and parallel output with latch functionality. One FPGA controls all 512 PIN diodes through a bank of shift registers and provides an output voltage of 0.8 or 0 V at the output of the shift registers [35]. This design allows parallel control of all diodes' switching states using minimal I/O ports (DS, STCP, SHCP and OE). The control circuit board is shown in Figure 6c. Any desired beamforming effects can be achieved by converting the MATLAB‐computed phase matrices into 8‐bit arrays and sequentially inputting them into cascaded shift registers. This method implements programmable beamforming manipulation through digital control.

**FIGURE 6:**
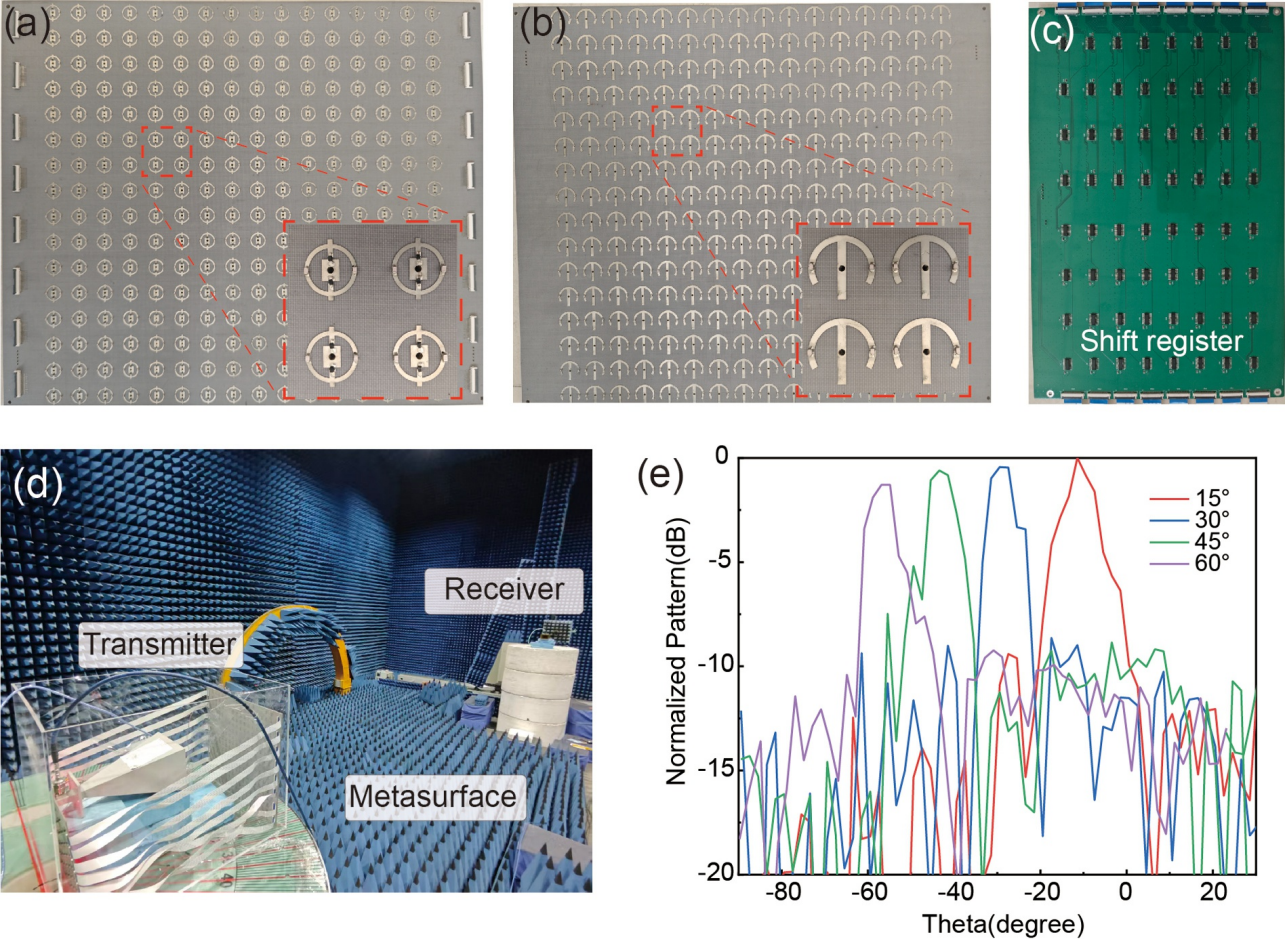
Top (a) and bottom (b) views of the RRTM prototype. (c) The shift register board. (d) Experimental setup. (e) The measured far‐field radiation pattern for beam angles of 15°, 30°, 45°, and 60° in the H‐plane at 8 GHz.

In fact, the voltage switching speed is very fast. When the clock frequency of the 74HC595 chip is 10 MHz, the time required for the serial input and parallel output of 512 bits of data is approximately 50 μs. The response time of the PIN diodes is on the nanosecond scale. Therefore, the total reconfiguration time, including both digital loading and diode switching, is still far below the millisecond level.

Figure 6d illustrates the measurement setup for the RRTM prototype. The control circuitry was housed within a custom‐made acrylic box, with the metasurface mounted on the right side and the control board positioned on the left. Ribbon cables connecting the circuitry and the array were secured at both ends to minimise interference with the feed horn. The feed horn was supported by foam and positioned at a focal distance of 350 mm. The radiation characteristics of the antenna were measured at 8 GHz using a far‐field measurement system. Because of limited measurement resources, the experimental test only involved controlling the RRTM with an FPGA to scan the beam in the H‐plane from 0° to 60°, as shown in Figure 6e. A comparison between the test results and the simulation outcomes reveals a high degree of similarity in the main lobe radiation patterns, with only minor discrepancies in beamwidth.

To verify the information transmission capability of the transmissive metasurface, we built a video transmitting radio system verification platform that achieves signal coverage at different reception angles through various encoding states. The experiment employed a universal software radio peripheral USRP‐LW B210 for baseband processing and modulation, along with up/down conversion devices to shift the baseband signal to an operational frequency of 8 GHz. As shown in Figure 7a, the transmitting horn antenna was placed 35 cm in front of the metasurface, and the receiving horn antenna was positioned at a 15° angle relative to the metasurface to verify its beam‐steering capability at 15°. The receiving side also used a USRP for down‐conversion, synchronisation and demodulation, ultimately recovering and playing the video on the receiving equipment [36, 37]. The screen pictures clearly demonstrate that the link can maintain stable video transmission even under beam‐steering conditions. Meanwhile, Figure 7b displays the corresponding 16‐QAM constellation diagram, where the symbol cloud maintains good clustering around the ideal constellation points, indicating that the transmissive metasurface can preserve high modulation quality and transmission reliability while participating in beam control. It should be noted that this verification was conducted for only a single angle. This is a proof‐of‐concept beam‐steered wireless‐link demonstration rather than a complete communication‐system characterisation. Therefore, since beam steering capability has already been verified previously, further experimental validation is not deemed necessary here.

**FIGURE 7:**
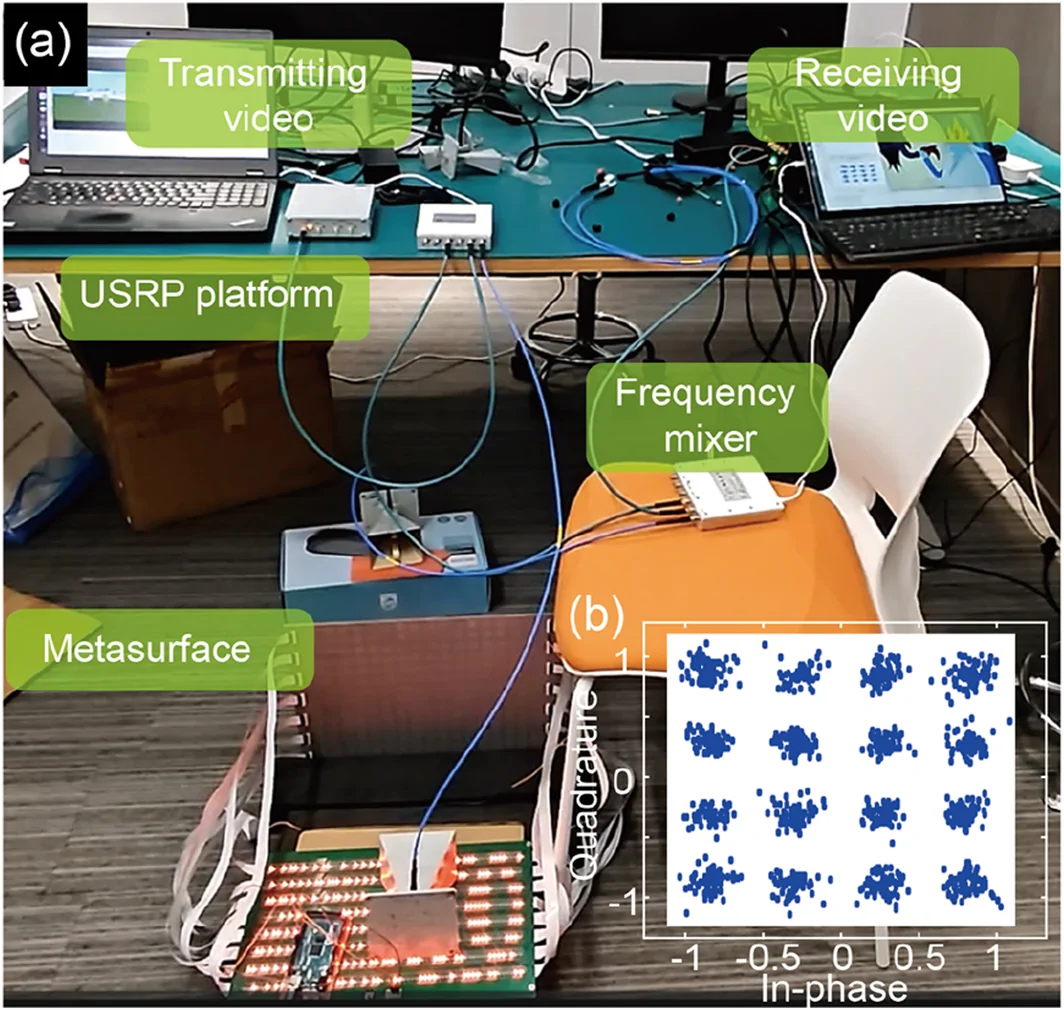
(a) Experimental set‐up of wireless communications at a 15° beam‐steering angle at 8 GHz. (b) 16‐QAM constellation.

### 2.3 Reflective Mode Operation

The beam scanning capabilities in the transmissive mode, achieved through phase compensation, have been demonstrated. Similarly, since states ‘11’ and ‘00’ exhibit a 180° phase difference around 7.7 GHz in the reflective mode, beam scanning effects for a reflectarray antenna can be achieved using analogous methods. This aspect is not elaborated further here. Instead, we introduce a more practically applicable scenario: backscatter enhancement. Dynamic retroreflection can be combined with RCS reduction modes, allowing for the implementation of corresponding functionalities by switching the states through encoding. It is important to note that while the optimal simulation frequency for the periodic unit is around 7.7 GHz, the optimal frequency shifts to 8 GHz for the actual prototype because of the finite size and manufacture deviation. Consequently, both transmission and reflection will be validated at 8 GHz in the following discussion.

The FPGA's control over all PIN diodes enables retroreflection of incident waves from all directions. Nevertheless, for the sake of experimental convenience, the H‐plane has been chosen for verification. Two methods exist for calculating the phase gradient for retroreflection, both utilising the generalised Snell’s law. One method involves calculating the incident angle from a known phase gradient, whereas the other calculates the ideal phase gradient based on the incident angle and then performs 1‐bit phase quantisation. The first method is introduced here. As depicted in Figure 8a,b, in cases where each column maintains the same state, the lateral periodic sequence is altered. In Figure 8a, every two columns constitute a period, with a phase difference of π between adjacent units. In Figure 8b, every four columns form a period, with an equal phase difference of π between adjacent units. When the incident electromagnetic wave frequency is 8 GHz, *λ* = 37.5 mm. Based on the derivation using the generalised Snell's law, the following Equation is obtained [38]:

(3) $$\sin\;\theta_{r}=\sin\;\theta_{i}+\frac{\lambda }{L_{x}},$$

where *L*_*x*_ represents the physical length of a period in the *x*‐direction. When retroreflection happens, $\theta_{r}=-\theta_{i}$, so:

(4) $$\sin\;\theta_{i}=-\frac{\lambda }{2L_{x}}.$$

**FIGURE 8:**
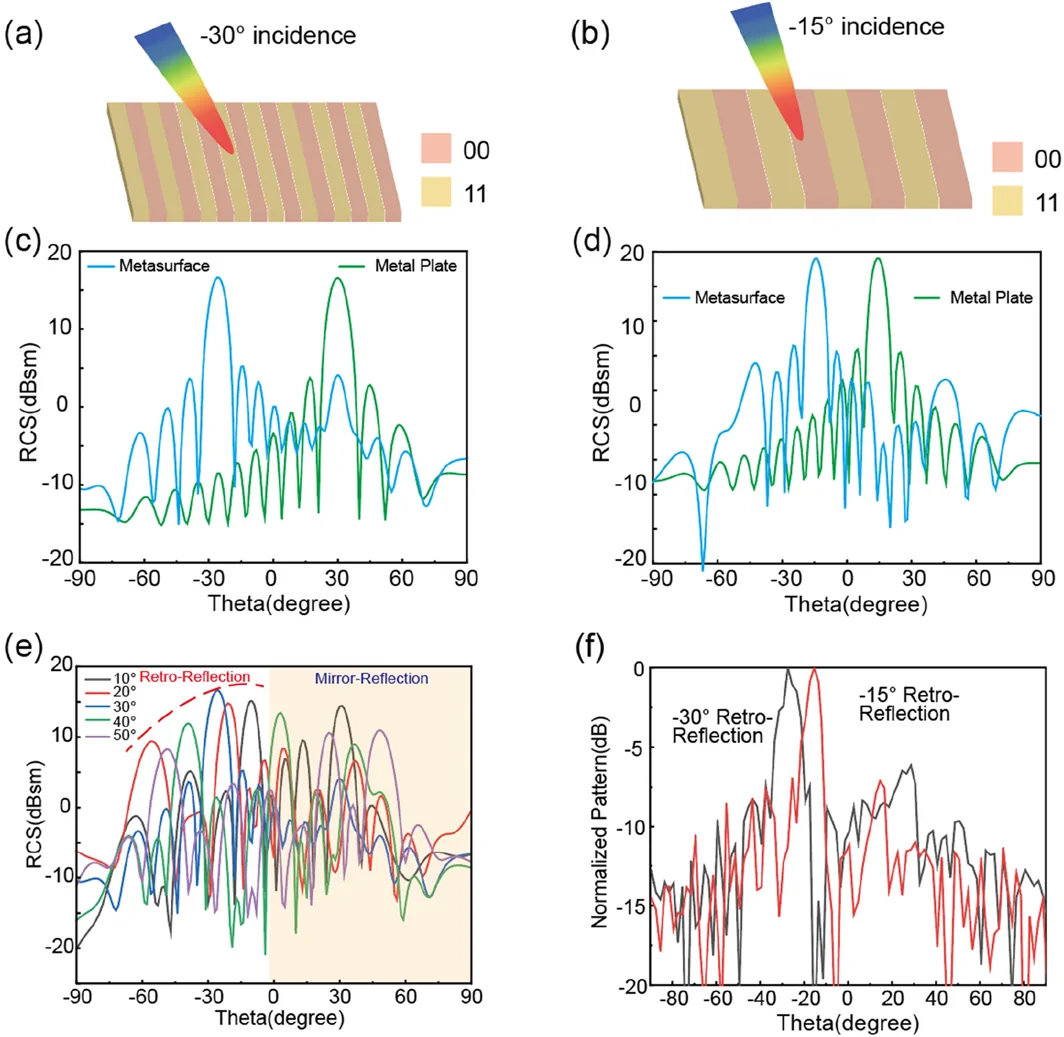
Electromagnetic waves incident at −30° (a) and −15° (b) on metasurface structures with different periodic sequences. (c) 2D far‐field scattering patterns of the metasurface and a metallic plate under −30° incident electromagnetic waves. (d) 2D far‐field scattering patterns of the metasurface and a metallic plate under −15° incident electromagnetic waves. (e) Retroreflection radiation patterns for −10°, −20°, −30°, −40°, and −50° calculated using quantised 1‐bit phase. (f) The measured normalised pattern of retroreflection for −30° and −15°. All of the above results are obtained at 8 GHz.

For Figure 8a, *L*_*x*_ = 36 mm, $\theta_{i}\approx -30{}^{\circ}$. For Figure 8b, *L*_*x*_ = 72 mm, $\theta_{i}\approx -15{}^{\circ}$.Consequently, Figure 8c,d represent the 2D radiation patterns of electromagnetic waves incident at −30° and −15°, respectively, corresponding to Figure 8a,b. It can be observed that the metasurface exhibits enhanced backscattering at the same angle as the incident wave, resulting in maximum gain at the angle of −30° and −15°. In contrast, a metallic flat plate exhibits specular reflection.

The second approach involves predefining the incident angle and calculating the phase gradient required for retroreflection:

(5) $$\frac{d\phi }{dx}=2k_{0}\;\sin\;\theta_{r},$$

where *k*_0_ is the wave number and *θ*_*r*_ is the retroreflection angle. Multiplying this gradient by the unit cell's position coordinates yields the continuous phase distribution for each unit cell. This distribution is then subjected to 1‐bit quantisation to obtain the target phase profile. The entire process, from calculating the phase gradient to obtaining the radiation pattern, can be accomplished through joint simulation using MATLAB and CST. Figure 8e illustrates the retroreflection radiation patterns for incident angles of −10°, −20°, −30°, −40° and −50°, calculated and simulated using the aforementioned equation. It can be observed that while the calculated phase gradient achieves the desired retroreflection in the retroreflective channel, significant 1‐bit quantisation errors lead to considerable sidelobes in the specular direction. This approach is therefore less precise and efficient than the first method. However, it is capable of achieving retroreflection at each specific angle, offering an 8–20 dB improvement compared to mirror reflection, thus satisfying the demand for continuous angular retroreflection.

### 2.4 Integrated Transmission‐Reflection Metasurface System

The dual‐mode operation (transmission and reflection) enables two distinct implementation schemes. First, a time‐division bidirectional beam scanning system can be designed, where transmission‐mode beam scanning is performed in one time slot and reflection‐mode scanning in another [39]. Alternatively, the 16 × 16 array can be partitioned through spatial encoding to achieve simultaneous transmission and reflection functionality. Leveraging the diode‐switched reconfigurability between transmission/reflection modes, we propose a four‐state coding strategy to unify both functionalities within a single metasurface architecture.

Whereas existing works have demonstrated partitioned modulation for independent reflection/transmission beam steering (with feed sources on one side), our design focuses on a pragmatic hybrid scenario: enhanced backscattering in reflection mode coupled with directionally scanned communication in transmission mode. Simple spatial partitioning (e.g., left‐half reflection/right‐half transmission) reduces effective aperture, causing beam broadening and higher sidelobes that degrade scanning precision. Our innovation lies in a checkerboard‐patterned metasurface where units adopt reflection mode when the sum of their row and column indices is even, and transmission mode when odd, as shown in Figure 9a.

**FIGURE 9:**
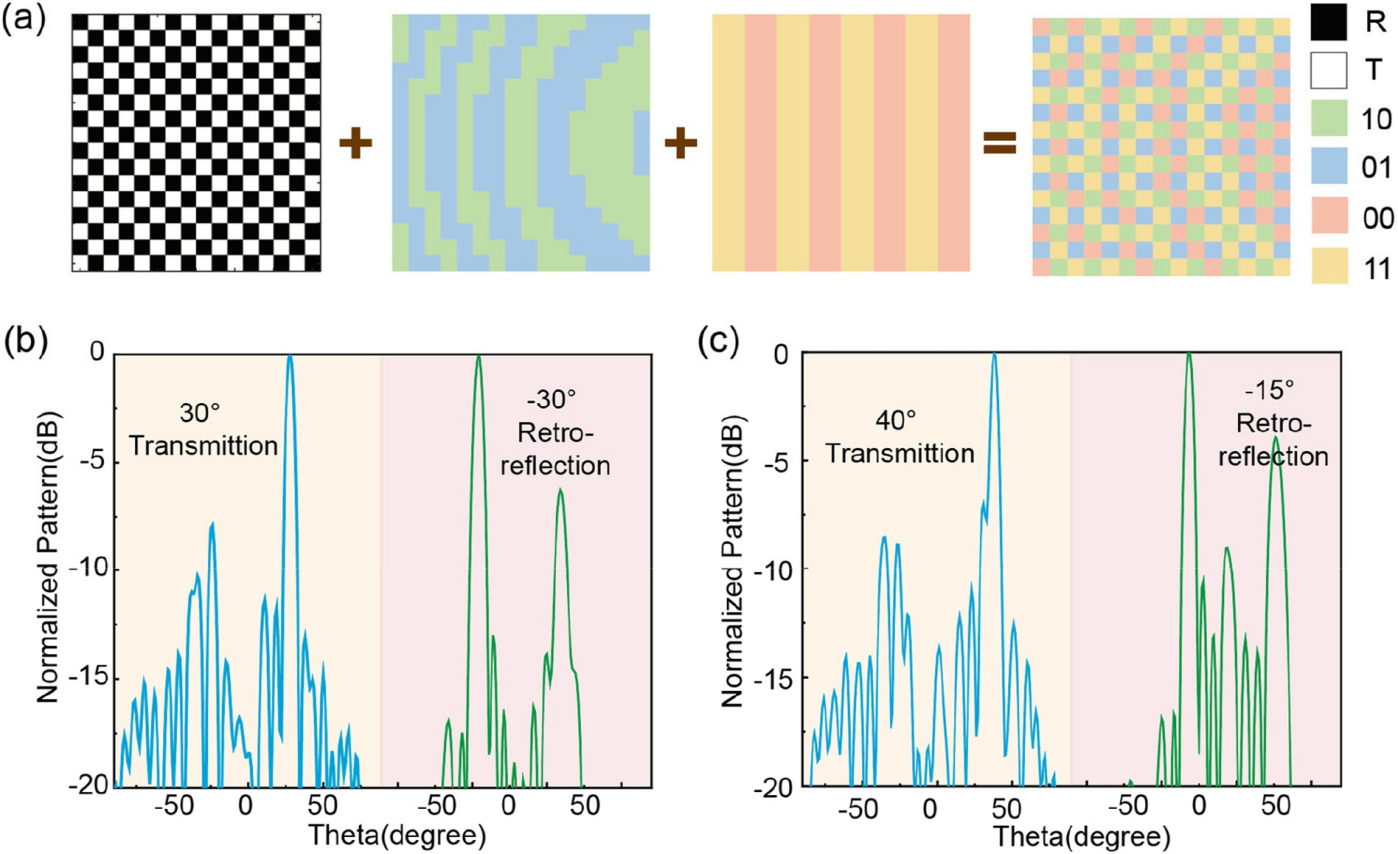
(a) Checkerboard‐patterned transmission and reflection coding distributions. (b) The simulated normalised far‐field scattering patterns of simultaneous transmission beam scanning at 30° and −30° retroreflection in the H‐plane. (c) The simulated normalised far‐field scattering patterns of simultaneous transmission beam scanning at 40° and −15° retroreflection in the H‐plane.

This configuration, which still incurs an effective‐aperture penalty but distributes each sub‐function over the full footprint, thereby mitigating beam broadening and making sure high‐precision beam pointing.

This configuration preserves full aperture utilisation for both modes, eliminating the trade‐off between functionality and radiation performance. Crucially, it maintains narrow beamwidths and suppressed sidelobes essential for high‐precision beamforming.

Figure 9b demonstrates an implementation of −30° retroreflection at the reflective terminal accompanied by simultaneous 30° beamforming at the transmissive terminal. As illustrated in Figure 9c, −15° retroreflection is achieved at the reflective terminal while generating 40° beamforming at the transmissive terminal. Both configurations exhibit favourable performance, collectively validating the metasurface's capability to simultaneously achieve retroreflection and transmit three‐dimensional beam scanning. These experimental results conclusively demonstrate the practical value of the proposed dual‐function metasurface supporting bidirectional concurrent operations.

Furthermore, the metasurface and its control circuitry exhibit low power consumption characteristics. Each diode requires less than 10 mA forward current with a turn‐on voltage of 0.8 V. Since only half of the diodes are activated in either transmission or reflection mode, the direct current power consumption of the metasurface array is calculated as 256 × 0.8 V × 10 mA = 2048 mW. However, additional power consumption (approximately 20–30 W) is required by the Arduino microcontroller and peripheral control circuits, including operational transistors, integrated chips and status‐indicating LEDs. The LED array serves as a visual interface for real‐time monitoring of metasurface operational states, which could potentially be eliminated in subsequent designs to optimise energy efficiency.

## 3 Conclusion

In this work, we have presented a programmable retro‐reflective/transmissive metasurface as a shared‐aperture platform for the decoupled co‐existence of backward scattering engineering and forward beamformed wireless links. The proposed four‐state PIN‐loaded meta‐atom enables dual‐channel 1‐bit phase control, where the ‘00/11’ states constitute a π‐phase pair in reflection and the ‘01/10’ states constitute a π‐phase pair in transmission, with good isolation between two functionalities, providing the fundamental conditions for decoupling. Enabled by an FPGA‐driven control architecture, a 16 × 16 prototype operating around 8 GHz supports rapid reconfiguration among (i) transmissive beam steering for forward link establishment, up to ± 60° (ii) angular‐adaptive retroreflection up to 50° and (iii) a shared‐aperture simultaneous configuration realised by an interleaved (checkerboard) coding strategy. The beam‐steered transmission was further verified by a wireless link demonstration with a stable 16‐QAM constellation, whereas the backward functionality was confirmed by the measured retroreflection/backscatter enhancement results. Overall, these results provide an experimentally grounded shared‐aperture route for integrating programmable scattering enhancement and transmissive communication on a single metasurface platform, and they establish a practical basis for future system‐level extensions.

## Author Contributions

**Shulei Zhang:** investigation, conceptualization, writing – original draft, writing – review and editing, formal analysis, software. **Ruichao Zhu:** investigation, writing – review and editing, visualization, funding acquisition. **Yuxiang Jia:** supervision, data curation, project administration. **Fan Wu:** software, formal analysis, project administration. **Xinqi Cai:** methodology, investigation, validation. **Zhihui Zhang:** methodology, software, data curation. **Dan Liu:** methodology, validation, formal analysis. **Yuxiao Guo:** project administration, writing – review and editing, validation, formal analysis. **Jiafu Wang:** funding acquisition, writing – review and editing, supervision, resources, project administration.

## Data Availability

The data that support the findings of this study are available from the corresponding author upon reasonable request.
